# An Overview of Acoustic-Based Interventions to Improve Motor Symptoms in Parkinson’s Disease

**DOI:** 10.3389/fnagi.2020.00243

**Published:** 2020-08-14

**Authors:** Jessie Siew Pin Leuk, Linette Li Neng Low, Wei-Peng Teo

**Affiliations:** ^1^Physical Education and Sports Science (PESS) Academic Group, National Institute of Education, Nanyang Technological University, Singapore, Singapore; ^2^Institute for Physical Activity and Nutrition (IPAN), School of Exercise and Nutrition Sciences, Deakin University, Geelong, VIC, Australia

**Keywords:** music therapy (MT), Parkinson’s disease, motor symptom (MDS-UPDRS-III), rhythm entrainment, acoustic therapy

## Abstract

Parkinson’s disease (PD) is characterized by motor and cognitive deficits that negatively impact on activities of daily living. While dopaminergic medications are used to attenuate motor symptoms, adjuvant therapies such as acoustic-based non-pharmacological interventions are used as a complement to standard drug treatments. At present, preliminary studies of acoustic-based interventions such as rhythmic-auditory stimulation (RAS) and vibroacoustic therapy (VAT) suggest two competing hypotheses: (1) RAS may recruit alternative motor networks that may bypass faulty spatiotemporal motor networks of movement in PD; or (2) the use of RAS enhances BG function through entrainment of beta oscillatory activities. In this mini review article, we discuss the mechanisms underlying the role of acoustic-based interventions and how it may serve to improve motor deficits such as gait impairments and tremors. We further provide suggestions for future work that may use a combination of RAS, VAT, and physical therapy to improve motor function in PD.

## Introduction

Parkinson’s Disease (PD) is the second most ubiquitous neurodegenerative disorder that affects approximately 1% of individuals aged over 60 (Reeve et al., 2014). The prevalence of PD increases with age, and with an aging population, the number of individuals with PD is expected to double in the next decade (Rizek et al., 2016). Individuals with PD exhibit both motor (tremor; rigidity; bradykinesia) and non-motor related symptoms (cognitive; neurobehavioural abnormalities; and sleep disorders) that stems from dopamine depletion in the substantia nigra within the basal ganglia (BG; Chaudhuri et al., 2006; Sveinbjornsdottir, 2016). While dopaminergic medications can effectively manage symptoms, drug-resistance and levodopa-induced dyskinesias are common side-effects following long-term use (Vorovenci et al., 2016). Adjuvant physical therapies such as acoustic-based interventions may therefore be efficacious in promoting wellness and maintenance of physical and cognitive health in PD.

Music is an effective mood relaxer (Magee and Davidson, 2002), beneficial in relieving anxiety, pain, and stress (Nilsson, 2008) at a low cost. Recent advancements in brain imaging have allowed us to revise how our brains respond to music (Hunt, 2015). An established view is that music induces adaptable changes in the auditory-motor network, that may facilitate functional recovery of movements, thus may be harnessed in the treatment of motor-related disorders (Sihvonen et al., 2017). Neuroimaging studies have further reported the recruitment of sensorimotor areas during music listening in the absence of movement (Gordon et al., 2018), and enhanced motor connections in stroke patients during the musical intervention (Altenmüller et al., 2009). Furthermore, studies suggested a close connection between auditory, BG (Nombela et al., 2013), and cerebellar networks (Thaut et al., 2009). Since music can modulate neural activities that are implicated in PD, it is plausible that acoustic-based intervention may represent a low-cost yet effective treatment for people with PD.

Indeed, acoustic-based interventions have previously been shown to improve gait parameters (see Table 1 for studies using acoustic-based intervention in PD). A common method of acoustic-based intervention used in neurorehabilitation in PD is rhythmic auditory stimulation (RAS) in which external auditory cues (i.e., beat of a metronome) are applied to synchronize or provide timing to the movement (Ghai et al., 2018). A typical RAS procedure starts with presenting beats of a metronome that match an individual’s baseline cadence. The metronome beats are then gradually adjusted to an optimal pace, and individuals are tasked to synchronize their steps with the beats. This process requires the entrainment of movements to external auditory cues; the latter involves the coordination of timing and movement sequencing (Thaut, 2005). This mini-review will attempt to discuss the common types of acoustic-based interventions used to improve motor symptoms associated with PD. Additionally, we will provide suggestions for future investigations and plausible mechanisms underlying the role of acoustics in normalizing the aberrant neurophysiology of PD.

**Table 1:** Effects of auditory therapy (stimulation and vibration) on motor symptoms of PD.

References	Subjects	Motor symptoms severity	Intervention and assessments	Outcomes	Conclusion
Buard et al. (2019)	Three PD patients (“on” state)	Y&H scale: 2–3 UPDRS III score: 23–52	Stimuli: Auditory cueing *via* metronome or beats produced by musical instrument (frequency: NS) Task: Bimanual exercises using keyboard, castanets and miscellaneous objects to strengthen fine motor muscles (3 times/week for 5 weeks) Assessments: UPDRS III, Grooved Pegboard Test, Finger-Thumbs Opposition Task were assessed pre and post-intervention period	Motor: Improved performance in overall motor assessments two out of three subjects improved in Grooved Pegboard and Finger-Thumbs Opposition Tasks Neurophysiology: Spectral analysis in primary motor and auditory cortices showed a simultaneous increase in evoked power in the beta-range, increased motor-auditory functional connectivity	Improvements in task performance were not consistent in all subjects but all of them benefitted in one or more fine motor function NMT-based motor rehabilitation enhances motor-auditory cortical activation in a synergic manner
Calabro et al. (2019)	Fifty Non-demented idiopathic PD patients (“on” state): 25 in RAS group and 25 in No RAS group	Y&H scale: 2–3	Stimuli: Auditory stimuli *via* music and the beats were emphasized with superimposed salient high-pitch bell sound (frequency: increase gradually up to 120 bpm through first 3–5 trials) Tasks: Walk on the treadmill with/without salient beats (GaitTrainer3), real-time feedback provided (5 times/week for 8 weeks) Assessments: Functional measures (FGA, UPDRS, BBS, Tinetti FES, 10MWT TUG, and GQI) were assessed at pre and post-intervention period Brain oscillation changes within the frontal, centroparietal, and temporal areas that were related to the gait cycle	Motor: RAS group showed greater improvements in overall gait quality, balance, number, length of strides and UPDRS scores compared to no RAS group A significant correlation between changes in connectivity measurements and gait Neurophysiology: RAS group showed significant changes in gait-related α and β ERS and ERD within frontal and centroparietal areas compared to no RAS group RAS group showed stronger entrainment of β oscillations between fronto-centroparietal and fronto-temporal connectivity compared to no RAS group	RAS improves overall gait quality, balance, number, and length of strides. Rhythmic entrainment shown in RAS group may indicate a restoration of internal timing mechanism *via* a compensatory network
Cochen De Cock et al. (2018)	Thirty-nine non-demented PD patients (“on” state): 22 in positive response (PR) group and 17 in Non-positive response (NPR) group Thirty nine matched healthy controls	Y&H scale: 2 ± 0.5 UPDRS III score: 24.3 ± 13.2	Stimuli: Auditory cueing *via* metronome and musical excerpts from four military marches (frequency: 10% faster than patient’s preferred cadence measured at pre-test) Task: Respond to the rhythmic stimuli and movement measured *via* 3D accelerometers and gyroscope PR groups were more musically trained, showed strong gait synchronization to the beat and better music perception compared to the non-positive response group (NPR).	Motor: PR group improved speed and stride length in contrast to the NPR group who showed worsening performance with rhythmic cues	While PD has timing and rhythmic deficits, patients in the PR group can still synchronize to beats Subject’s rhythmic abilities and musical training may affect their response to auditory cueing
			Assessments: Gait parameters (cadence, velocity, stride length, variability, coordination) and rhythmic/musical abilities (BAASTA, beat alignment test, finger tapping task, and Gold-MSI) were assessed pre and post auditory cueing
Harrison et al. (2018)	Thirty non-demented PD patients (“on” state) 30 young controls aged 18–35 Thirty older controls aged ≥50	UPDRS III score: 24.9 ± 10.27	Stimuli: Internal cueing *via* singing aloud and walking to the beat of their singing. External cueing *via* music playing and walking to the beat of the song (frequency: patient’s preferred cadence measured at baseline) Task: Forward and backward walking trials (5 m GAITRite Walkway) in non-cued and cued conditions Assessments: Gait parameters (velocity, cadence, stride length, variability)	Motor: PD exhibits greater improvements compared to controls. During internal cueing (singing), improved gait velocity, cadence and stride length in backward walking, and reduced gait variability in both forward and backward walking During external cueing (music playing), minimal improvement in gait characteristic and higher gait variability compared to internal cueing	Auditory cueing improves gait in PD, particularly in backward moving (more challenging gait situation) Internal cueing *via* singing may be more beneficial to gait compared to external cueing
Murgia et al. (2018)	Thirty-two non-demented PD patients (“on” state): 16 in ecological RAS group and 16 in the artificial RAS group	Ecological RAS group- Y&H scale: 1.5–2.5 UPDRS III score: 18.0 ± 9.1 Artificial RAS group- Y&H scale: 1.5–3 UPDRS III score: 20.2 ± 9.6	Stimuli: Ecological RAS *via* footsteps recording that was chosen from a database.Artificial RAS *via* metronome sounds (frequency: personalized by comparing cadence in matched healthy individuals and the patient’s baseline cadence) Task: Walk along 10 m walkway while listening to either ecological or artificial RAS soundtrack (45 min/session, 2 times/week for 5 weeks) Assessments: Gait parameters (speed, length width, cadence, stance, swing, double support phase duration) and clinical measurements (i.e., FES, FOGQ, Y&H scale) were assessed with a motion capture system at pre, post and 3 months after the intervention period	Motor: RAS type showed no comparable differences. Improved overall gait and clinical measures during the post- and follow-up intervention. Exploratory analyses for separate groups showed Spatio-temporal gait improvements in ecological RAS group only	Ecological and artificial RAS are equally effective in improving gait and clinical assessment scores
Benoit et al. (2014)	Fifteen non-demented idiopathic PD patients (“on” state) 20 matched healthy controls	Y&H scale: 2 ± 0.7 UPDRS score: 37.7 ± 18.8	Stimuli: Auditory cueing *via* songs played without lyrics and the beats were emphasized with superimposed salient high-pitch bell sound (frequency: ± 10% of patient’s preferred cadence measured at first session)	Motor: Increased stride length during the post- and follow-up intervention Improved performance and ability to detect changes in synchronization task, hand tapping task, duration discrimination and detection of misaligned beats	Auditory cueing improves perceptual and motor timing beyond gait
			Task: Walk to the beats and continue walking when the beat stopped, including a stop-and-go phase. Subjects were not explicitly instructed to synchronize footsteps to the beat of the music (3 times/week for 1 month) Assessments: Perceptual and movement tasks (BAASTA) were assessed pre, post and 1 month after the intervention period
Kadivar et al. (2011)	Sixteen non-demented idiopathic PD patients (“on” state): 8 in RAS groups and 8 in No RAS group	Y&H scale: 2–4 UPDRS III score: RAS group- 27.1 ± 4.1 No RAS group- 27.0 ± 3.8	Stimuli: Auditory cueing *via* “cluck,” “ding” and “soft cork” sounds (frequency: patient’s preferred cadence determined by averaging first five trials, ±10%, ±20%) Task: Multidirectional step training with RAS at different frequencies. No RAS group performed self-selected, internally paced stepping (3 times/week for 6 weeks) Assessments: Functional measures (DGI, TUG, UPDRS, FOGQ, Tinetti gait and balance) were assessed pre, post and at different follow-up time points (1, 4, 8 weeks after post-intervention)	Motor: RAS group showed greater improvements in DGI scores compared to no RAS group Improvements in other functional measures were comparable in both groups RAS group maintained improvements in gait and balance longer than No RAS group	Step training with RAS improves functional gait and balance that could maintain over 8 weeks
Rochester et al. (2009)	Nine non-demented idiopathic PD patients with a modest degree of cognitive impairment	Y&H scale: 3 ± 2.5–3 UPDRS score: 44 ± 35.5–47 Modified dyskinesia score: >2 FOGQ scores: 11 ± 9–15.5 Tinetti gait and balance scale: 19.78 ± 4.41	Stimuli: Metronome (frequency: patient’s preferred cadence determined by averaging first three trials) Task: Walk only task on GAITRite and dual-task (walk while carrying a tray with two water-filled cups) with or without a cue. Cue either focusing on the temporal parameter (step in time to the beat) or spatiotemporal parameter (take a big step in time to the beat) Assessments: Gait parameters (walking speed, stride amplitude, cadence, step time, double limb support time, variability), UPDRS III	Motor: Cue focusing on spatiotemporal parameter showed great improvements in walking speed, stride amplitude and cadence in both single and dual-task Cue focusing on temporal parameter showed improvements in cadence	Auditory cueing (especially cueing focusing on step length while being prompted to maintain cadence) improved motor symptoms in PD patients with mild cognitive impairment
Fernandez del Olmo et al. (2006)	Nine non-demented idiopathic PD patients (“on” state): 4 akinetic-rigid dominant and five tremor dominant five matched healthy controls	Y&H scale: 1–2.5 UPDRS score: 12–45	Stimuli: Auditory cues *via* metronome (frequency: 60, 90, 120, 150 bpm) Task: Walk along 30 m walkway while performing specific increasingly complicated tasks in two conditions: reproduce walking speed after listening to metronome playing at different frequencies, synchronize walking with the presence of metronome playing at different frequencies (1 h/day, 5 days/week for 4 weeks) Assessments: Gait parameter (velocity, length, cadence, variability) and finger tapping performance were assessed pre and post-intervention period	Motor: Interval time between taps and between steps was more regular after the intervention period Other gait parameters did not reach significant improvements after the intervention Neurophysiology: PET analysis showed increased glucose uptake in the right anterior lobule of the cerebellum, dentate nucleus near the midline and right temporoparietal conjunction after training	Auditory cueing improves temporal variability of gait and finger tapping, along with increase metabolism in sensorimotor areas which indicate access to cerebellar projections to compensate for the damage BG
King et al. (2009)	Forty non-demented idiopathic PD patients (“on” state): 20 slow/rigid dominant and 20 tremors dominant	Not specified	Stimuli: Whole-body sound wave vibration *via* psychoacoustic chair (five series lasting 1 min each with 1-min rest in between, frequency: 27–113Hz) Task: Close eyes and relax during vibration. Walking task only (GAITRite) to assess gait. Assessments: UPDRS, gait parameters (velocity, step length), grooved pegboard performance were assessed at pre, post vibration and after a control rest period	Motor: The dominant Symptom category showed no comparable differences. Decreased rigidity and tremor, increased step length, improved speed on pegboard task (corresponding with reduced bradykinesia) after vibration Other UPDRS measures for posture, leg agility and sitting to standing scores did not reach significant improvements	The patient’s dominant symptom does not affect the treatment outcome. Vibration therapy improves the primary motor symptoms of PD (rigidity, tremors, and bradykinesia)
Haas et al. (2006)	Sixty-eight non-demented idiopathic PD patients (“on” state)	Y&H scale: 2–4 UPDRS score: 29.9 ± 11.9	Stimuli: Whole-body random unsynchronised vibration delivered to the feet (5 series lasting 1 min each with 1-min rest in between, frequency: mean of 6 Hz) Task: Standstill on a platform (ZEPTOR) with knees slightly bent Assessments: UPDRS motor scores and subscales were assessed at pre and post vibration	Motor: Improved overall UPDRS motor scores by 16.8% on treatment group after vibration while the control group showed non-significant changes Greatest improvements in tremor (25%) and rigidity (24%) scores, while gait (15%), posture (15%) and bradykinesia (12%) subscales showed a slight improvement	Vibration therapy is experienced by the whole-body and improves the motor symptoms of PD (rigidity, tremors, gait, posture, and bradykinesia)

*Abbreviations: PD, Parkinson’s Disease; NMT, Neurologic Music Therapy; RAS, Rhythmic Auditory Stimuli; ERS, Event-related Synchronisation; ERD, Event-related desynchronization; BG, Basal Ganglia; bpm, beat per minute; H&Y scale, Hoehn and Yahr scale; UPDRS, Unified Parkinson’s disease rating scale; BAASTA, Battery for the Assessment of Auditory Sensorimotor and Timing Abilities; Gold-MSI, Goldsmiths Musical Sophistication Index; FES, Falls Efficacy Scale; FOGQ, Freezing of Gait Questionnaire; FGA, Functional Gait Assessment; BBS, Berg Balance Scale; 10MWT, 10 Meter Walk test; TUG, Timed Up and Go; GQI, gait quality index; DGI, Dynamic Gait Index*.

## Mechanisms Underpinning RAS

In RAS, the application of external auditory cues serves as a time reference to regulate walking pace to external rhythm, allowing temporal expectations (anticipation of the next steps) to occur (Rohenkohl et al., 2012; Nombela et al., 2013). Motor symptoms in PD are likely due to faulty temporal and spatial mechanisms, necessary for coordinating and initiating structured movements (Jones et al., 2008). Therefore, auditory cues can act as an “internal clock” to stabilize aberrant internal rhythm in PD individuals *via* neural entrainment. An fMRI study by Grahn (2009) found that when the internal generation of beats was needed, the BG, specifically putamen activity was lowered with the presence of strong external beats. This suggests that the putamen may play a crucial role in mental beat prediction and generating accurate motor execution pace.

**Figure 1:**
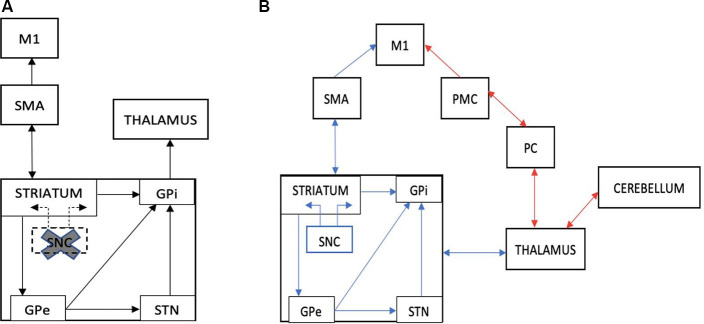
**(A)** An illustration of the striato-thalamocortical motor loop that is impaired in Parkinson’s disease (PD) due to dopamine depletion within the SNC of the basal ganglia (cross and dotted lines). **(B)** An illustration of The Compensation Hypothesis, which suggests recruitment of the cerebello-thalamocortical pathway that bypasses the BG (red lines). Alternatively, The Restoration Hypothesis suggests a facilitation effect on the striato-thalamocortical pathway, specifically facilitating dopaminergic function in the basal ganglia, (blue lines). M1, primary motor cortex; SMA, supplementary motor cortex; PMC, premotor cortex; PC, parietal cortex; SNC, substantia nigra pars compacta; GPe, external globus pallidus; GPi, internal globus pallidus; STN, subthalamic nucleus (adapted from Fujii and Wan, 2014).

Besides its role in time perception to regulate movement, the BG mediates the interaction between auditory and motor systems during rhythm perception. This results in the engagement of motor areas (i.e., supplementary and premotor areas) when people attend to rhythms (Zatorre et al., 2007; Grahn, 2009; Grahn and Rowe, 2013; Ashoori et al., 2015; Raglio, 2015), which underpins the concept of RAS. Herein, we ask if RAS can potentially confer improvement when key brain areas required for rhythm and/or motor timing perception are impaired? The answer may be two-fold, with distinct hypotheses: Compensation and Restoration (Figure 1).

## The Compensation Hypothesis

The compensation theory suggests that while the BG is impaired in PD, other cortical structures may execute the functions of rhythm perception typically performed by the BG. Acoustic rhythms may reinforce alternate networks, such as the cerebello-thalamocortical circuitry, to modulate neural entrainment and enhance motor functioning (Schwartze et al., 2011; Nombela et al., 2013). When movements were externally cued, studies have shown increased activation within the lateral premotor cortex (PMC), and cerebellar connections to motor cortices (Sen et al., 2010; Todd and Lee, 2015), which effectively bypasses the impaired striato-thalamocortical circuitry. Moreover, Fernandez del Olmo et al. (2006) reported elevated glucose uptake in the cerebellum of individuals with PD post-RAS training using positron emission tomography (PET) concomitant to reductions in the variability of gait and finger-tapping tasks. The enhanced cerebellar activity after a month of RAS may imply that cerebellar pathways have been recruited to compensate for the impaired BG-cortical circuitry.

## The Restoration Hypothesis

In contrast, some reviews suggest that acoustic rhythms may facilitate impaired BG network in individuals with PD (Nombela et al., 2013; Raglio, 2015) rather than bypassing it as suggested by the Compensation Hypothesis. Beta activity is excessively synchronized in individuals with PD leading to motor dysfunctions (Little and Brown, 2012; Sharott et al., 2014). The mechanistic rationale of RAS in PD is therefore to normalize beta-band coupling of the motor network, which may provide similar effects to current treatments (i.e., deep brain stimulation and dopaminergic therapy) without surgery or medication (Ghai et al., 2018).

The Restoration Hypothesis is supported by a study conducted by Woerd et al. (2014), which suggested that RAS was beneficial only if there was an increase in modulation depth of beta oscillatory activity, defined as the difference between event-related synchronization and desynchronization (ERS/ERD). Their study showed that relative to controls, individuals with PD had lesser ERD before the cue, with greater ERD after cue, probably due to compensation. Therefore, increases in beta ERS reflected the involvement of the BG as a beat predictor in rhythm processing, whereas increases in beta ERD is likely to reflect compensatory mechanisms. The findings from Woerd et al. (2014) showed that gains in modulation depth of beta activity are comparable amongst patients and controls. Moreover, the gains are entirely due to increases in synchronization, conferring increased predictive movement-related beta power suppression. This indicates that the BG is involved in rhythm and motor timing perception in individuals with PD and that RAS facilitates this process.

Overall, both hypotheses have valid supporting evidence and at present, we have yet reached consensus about the neuroplastic changes associated with RAS. Future studies building on theory are thus encouraged to explore whether RAS facilitates movements by bypassing or/and facilitating the impaired BG network in PD.

## Neurochemical Effects of RAS

On top of neural network reorganization, studies have highlighted the neurochemical effects of RAS (Chanda and Levitin, 2013; Sihvonen et al., 2017; Cochen De Cock et al., 2018). Music listening not only activates emotional networks but also stimulates dopamine production in the striatal system (Salimpoor et al., 2011). Emotions and physical movements are closely intertwined (Molnar-Szakacs and Overy, 2006), hence there are suggestions that motor improvements following music intervention may be a result of emotional reactions mediating the dopaminergic activity of the BG motor loop (Salimpoor et al., 2011; Morris et al., 2019).

A recent PET study by Koshimori et al. (2019) explored RAS-induced dopamine responses in the BG of healthy participants while they performed finger-tapping tasks. They reported that RAS significantly decreased the binding potential relative to the non-displaceable compartment (BP_ND_) variability in the BG. Consequently, this led to a significant decrease in DA response in the left ventral striatum (VS), an area that plays a role in motivation and reward processing (Liljeholm and O’Doherty, 2012). The authors further explained that this decreased in DA response with RAS was associated with lesser motivational and attentional requirements while attending to the task, which could be beneficial in dopamine-deficient patients. This study, therefore, provided insights about the potential role of RAS to modulate dopamine responses in PD and warrant future studies with PD patients.

Morris et al. (2019) surveyed the music responses of people with and without PD. They observed that PD patients had a diminished connection between music and action, compared to controls who perceived music as a reward and felt motivated to sing or dance along. Motor responses during reward anticipation are mediated by the release of DA in the VS (Elliott et al., 2004), which is depleted in individuals with PD and may lack the motivation to act. If the results in Koshimori et al. (2019) study were taken into account, RAS may, therefore, be beneficial to individuals with PD as this intervention could lead to reduced DA response in the VS. Additionally, music mitigates motor impairments in PD by activating the emotional-limbic-network and inducing dopamine secretion within domains of reward and motivation (Chanda and Levitin, 2013), a process akin to taking dopamine medications. The reward centers remain connected to motor areas, whilst dopamine serves as reinforcement to motivate repeat behaviors individuals find pleasurable.

While the potential of RAS has been widely discussed, most studies do not specify the details of RAS, such as the type of acoustics employed and the intervention duration. Some studies presented metronome beats on their own (Dalla Bella et al., 2017; Rose et al., 2019), whereas others embedded beats within music selected by the patient (Thaut et al., 1996), or by therapists (McIntosh et al., 1997). Leow et al. (2014) proposed the use of familiar music/music with high beat salience during RAS therapy for better improvements in gait parameters, because less cognitive demand is required to entrain movement with familiar and strong beat structures. Furthermore, music enjoyment elicits an emotional response and serves as a good motivator during intervention programs. Acoustic-based intervention is also easier to incorporate into a patient’s lifestyle as opposed to other physical therapies. A suggestion for future investigation would be to explore how different genres of music affect movement and emotional states of PD patients. They may also investigate optimal duration (number of sessions) alongside long-term effects of RAS therapy when patients are off medication, to observe if therapeutic effects are retained.

## Sound Vibration Reduces Tremors

The bulk of acoustic-based intervention studies on PD report improvements in bradykinesia and speech functions using RAS. However, tremors are often the first symptoms in PD to be noticed. These can affect the hands, mouth, and limbs (Jankovic, 2008), impacting activities of daily living (Bhidayasiri, 2005). Very limited studies to date have explored how acoustic-based interventions help reduce or control tremors.

To our best knowledge, tremor reduction is evidenced in studies applying Vibroacoustic therapy (VAT; see Table 1). VAT delivers passive low-frequency sound vibrations (20–100 Hz) in contrast to RAS, the latter, an active form of acoustic therapy. In clinical settings, VAT supports the idea that patients should not only listen to music in isolation but sense vibrations as well (Warth et al., 2015). The VAT involves transducers embedded into chairs or mattresses, which send vibrations to and through the body. According to Punkanen and Ala-Ruona (2012), the best combination for VAT is a combination of music, sound waves and therapeutic interaction, which allows therapists to address a patient’s mental and cognitive states, alongside motor function.

Skille and Wigram (1995) demonstrated that interventions involving music plus vibrations (40 and 55 Hz) are most effective in enhancing/supporting movement while reducing muscle tension in physically disabled persons, in contrast to music as a solo acoustic therapy, indicating the importance of vibrations in neurorehabilitation. Studies that have implemented a combination of music and vibration in their intervention procedures include Chesky and Michel (1991), who reported vibroacoustic frequencies between 60–600 Hz to provide optimal pain relief; while Wigram (1996) reported decreased muscle tension, resulting in movement improvements in patients with cerebral palsy, post-intervention. Zheng et al. (2009) only applied low-frequency sound wave stimulation to frail, elderly participants, who later showed enhanced mobility. Nonetheless, many aspects of VAT are not clearly defined, and no agreement has been established regarding the necessity for music during VAT procedures. As such, the following section aims to discuss the individual roles of vibration and music in VAT, highlight studies that have applied vibration to reduce tremors in PD, and clarify possible mechanisms involved.

## The Relaxation Hypothesis

Studies that delivered vibrations to a single muscle (Jöbges et al., 2002) the entire body (Haas et al., 2006) or *via* physio-acoustic chair (King et al., 2009) have demonstrated some level of efficacy in reducing tremors in individuals with PD. These studies further suggest that vibrations applied to manipulate local sensory feedback to the muscles, which decrease tremor frequency. One hypothesis for its effect is related to relaxation, which is based on the concepts of resonance, such that every part of our body has its natural frequency (Punkanen and Ala-Ruona, 2012). VAT frequencies presented *via* vibroacoustic chair may cause entrainment of our natural frequencies to resonate with external frequencies, resulting in increased blood circulation, enhanced metabolism, and decreased tension in muscles (Zheng et al., 2009; Punkanen and Ala-Ruona, 2012).

Tremors in individuals with PD are exacerbated when they are mentally and emotionally stressed (Schlesinger et al., 2009; Buhmann et al., 2018), and conversely reduced during mental relaxation; disappearing in sleep (Poliaková and Králová, 2015). The key to curtailing tremors may be to provide patients with a relaxed state of mind (Punkanen and Ala-Ruona, 2012). Zach et al. (2017) measured tremor intensity and variability while PD patients were performing a mental arithmetic task in ON and OFF L-Dopa medication. They found that cognitive stress reduced the effects of the medication on resting tremors, which highlights the importance of relaxation. Since the purpose of VAT is to achieve mental and muscular relaxation, adding a component of music has the potential to enhance relaxation, whilst encouraging patients to receive therapy, providing the music to be chosen correctly. Therefore, the type of music employed in acoustic therapy should be carefully determined because certain musical genres may at times be distracting, causing irritation or triggering unpleasant memories (Punkanen and Ala-Ruona, 2012). Future studies should aim to investigate the role of music in VAT by comparing therapeutic effects between vibration only and vibration plus music. Also, neural factors accompanying VAT have not been extensively explored. It is debatable whether tremor reduction after VAT is merely due to a state of relaxation, or the entrainment of thalamic oscillations.

Besides the relaxation hypothesis, Punkanen and Ala-Ruona (2012) have provided two other theories to explain the possible mechanisms underlying VAT. The first suggests low-frequency sound stimulates pain inhibitory mechanisms (i.e., Pacinian corpuscle), which mitigates pain impulse to the brain. In other words, VAT may be an effective pain reliever because inhibitory interneurons are activated in the process (Janzen et al., 2019). The second hypothesis is Jindrak postulate. Since the brain does not have a lymphatic drainage system, vibrations through it may assist the removal of waste molecules *via* diffusion through intercellular spaces (Skille and Wigram, 1995). However, these hypotheses do not explain how VAT is beneficial for relieving PD symptoms. A recent paper by Janzen et al. (2019) suggests vibroacoustic stimuli mimic brain stimulation (e.g., during transcranial electrical stimulation, wherein brain oscillations are induced), which in turn regulate thalamocortical dysrhythmias and enhance functional connectivity of the pain network in fibromyalgia patients. Based on this concept of neural entrainment, we may speculate that the delivery of rhythmic entrainment and sound vibration achieved with VAT can regulate abnormal neural oscillatory activity in PD (Teo et al., 2017). This hypothesis warrants further investigation to elaborate on the effects of vibroacoustic stimuli on neural oscillation and network connectivity.

## Conclusion

In summary, there is growing evidence to support the beneficial effects of acoustic-based interventions in the form of music; rhythmic auditory cues; sound vibrations, or a combination of the three, in people with PD. Different PD symptoms can thus be treated with varying procedures such as RAS, which motivate the speed of movements, while sound vibration reduces involuntary shaking.

Herein, we propose directions for future research. If music serves as a relaxation tool to relieve symptoms, can individuals with PD habituate this form of relaxation to control their tremors through practice? As discussed, music plus vibration is far beneficial than either element alone; albeit systematic evidence to support this approach is necessary. Finally, the long-term effects of acoustic-mediated therapy in PD should be explored concerning its parameters (i.e., treatment duration; frequency; and type of music, where applicable) within each intervention. Systematic, longitudinal studies will greatly advance our understanding of music’s applicability in neurorehabilitation.

## Author Contributions

JL: manuscript conceptualization, writing, and editing. LL and W-PT: manuscript writing and editing.

## Conflict of Interest

The authors declare that the research was conducted in the absence of any commercial or financial relationships that could be construed as a potential conflict of interest.
